# Effects of sodium hypochlorite concentrations and irrigant activation on postoperative pain after single-visit root canal treatment in asymptomatic molars with chronic apical periodontitis: a randomized clinical trial

**DOI:** 10.1186/s12903-026-09567-x

**Published:** 2026-08-11

**Authors:** Ecem Karakoyunlu, Kübra Gürler, Koray Yilmaz

**Affiliations:** Faculty of Dentistry, Department of Endodontics, Cukurova University, Adana, Adana 01330 Turkey

**Keywords:** Activation, EDDY, Irrigant concentration, Passive ultrasonic irrigation, Postoperative pain, Sodium hypochlorite

## Abstract

**Background:**

This randomized, single-blind clinical trial evaluated whether sodium hypochlorite (NaOCl) concentration and irrigant activation influence postoperative pain after single-visit root canal treatment in molars with chronic apical periodontitis.

**Methods:**

One hundred twenty asymptomatic patients were randomly allocated to six groups (*n* = 20) in a 2 × 3 factorial design (NaOCl concentration: 2.5% vs. 5.25% and nonactivation [NA], EDDY [VDW; Munich, Germany], or passive ultrasonic irrigation [PUI]). Postoperative pain was recorded using a 10-cm visual analog scale (VAS) at 6, 12, 24, 48, and 72 h. Repeated VAS measurements were analyzed using a linear mixed-effects model with an autoregressive AR (1) covariance structure, including fixed effects for concentration, activation, time, prespecified interactions, and age as covariates; prespecified simple effects at 6 h were examined with Bonferroni adjustment.

**Results:**

VAS scores decreased significantly over the course of postoperative assessments in all groups (time effect, *P* < .001). In the overall model, the activation technique had a significant effect on postoperative pain (*P* = .011), whereas the NaOCl concentration did not (*P* = .529). Significant concentration × activation (*P* = .037) and activation × time (*P* < .001) interactions were also observed. At 6 h, the only pairwise difference that remained significant after Bonferroni adjustment was between NA and PUI (mean difference 1.546; *P* < .001), whereas the comparisons between NA and EDDY and between EDDY and PUI were not statistically significant. Analgesic intake (18.3%) was analyzed as a secondary outcome using patient-level logistic regression. The NaOCl concentration was not associated with analgesic intake (*P* = .972). In contrast, compared with those in the PUI group, participants in the EDDY group had significantly greater odds of analgesic intake than those in the PUI group (OR = 4.022; *P* = .036).

**Conclusions:**

Overall, under this clinical protocol, the activation modality appeared to have a greater influence on early postoperative pain than the NaOCl concentration alone did. However, postoperative pain scores remained generally low across all groups, suggesting that the clinical significance of these differences should be interpreted with caution.

**Trial registration:**

Current controlled trials NCT07033247 registered on 13 June 2025. Retrospectively registered.

## Background

Postoperative pain following endodontic treatment is an undesirable outcome for both clinicians and patients, whose reported incidence ranges from 3% to 58% [1]. There are various opinions regarding the frequency of postoperative pain in teeth with vital versus nonvital pulps; however, several studies have reported a higher incidence of postoperative pain in teeth with nonvital pulps [2, 3]. In teeth with necrotic pulp, bacterial infection is considered the primary etiological factor of apical periodontitis. It has been suggested that a high bacterial load may exacerbate inflammatory processes and that residual bacteria within the root canal system may lead to increased pressure in periapical tissues [4]. During root canal shaping in teeth with chronic apical periodontitis, extrusion of intracanal microbial content through the apical foramen may reactivate chronic lesions and subsequently result in postoperative pain [5].

Root canal irrigation plays a crucial role in enhancing the effectiveness of endodontic treatment by facilitating the removal of organic and inorganic debris from areas inaccessible to mechanical instrumentation during canal shaping [6]. Sodium hypochlorite (NaOCl) is the most commonly used irrigant owing to its potent antimicrobial and antibiofilm properties, as well as its ability to dissolve organic tissue, and it is available at concentrations ranging from 0.5% to 6% [7]. High NaOCl concentrations increase antimicrobial efficacy and tissue dissolution but may increase cytotoxicity if apically extruded, making the concentration–pain relationship clinically relevant [8]. Its clinical performance is also influenced by irrigant activation, which improves irrigant exchange and penetration into complex root canal anatomy [9].

Irrigant activation improves irrigant exchange and penetration by altering intracanal fluid dynamics, which may also influence postoperative pain through the apical extrusion of irrigants or debris. Experimental studies have demonstrated that different activation systems can produce different amounts of apically extruded debris, supporting the biological plausibility that activation modality could influence postoperative symptoms [9].

Passive ultrasonic irrigation (PUI; VDW Ultra, VDW, Munich, Germany) enhances irrigant effectiveness through acoustic streaming and cavitation generated by an ultrasonically activated tip placed within the canal [10, 11]. Sonic activation systems such as EDDY (VDW; Munich, Germany) use flexible polymer tips to improve irrigant movement and penetration within the root canal system [12]. EDDY operates with three-dimensional oscillatory motion, generating fluid agitation and streaming, and its flexible design facilitates its use in curved canals [13, 14]. Although both techniques aim to improve irrigant exchange and debris removal, their differing hydrodynamic characteristics may influence apical extrusion and postoperative outcomes.

Although postoperative pain has been investigated in relation to irrigation protocols, evidence regarding the combined effects of NaOCl concentration and irrigant activation in molars with chronic apical periodontitis remains limited. Therefore, this randomized controlled clinical trial investigated whether 2.5% versus 5.25% NaOCl combined with nonactivation (NA), EDDY, or passive ultrasonic irrigation (PUI) influenced postoperative pain following single-visit root canal treatment in molars with chronic apical periodontitis. Given the 2 × 3 factorial design, the primary objectives were to estimate (i) the main effect of NaOCl concentration, (ii) the main effect of activation technique, and (iii) the concentration-by-activation interaction on postoperative pain. Accordingly, the null hypotheses were that (1) postoperative pain does not differ by NaOCl concentration, (2) postoperative pain does not differ by activation technique, and (3) there is no interaction between NaOCl concentration and activation technique in terms of postoperative pain.

## Methods

This study was designed as a single-blind, randomized controlled clinical trial conducted at the Department of Endodontics, Faculty of Dentistry, Cukurova University. Ethical approval was obtained from the Clinical Research Ethics Committee of the Cukurova University Faculty of Medicine (Reference No: 149/2024/11). The trial was conducted between December 2024 and April 2025 and included 120 patients who met the predefined inclusion criteria. The study protocol was registered on ClinicalTrials.gov (NCT07033247) on February 11, 2025, after patient recruitment began. The study design, eligibility criteria, outcomes, and statistical analysis plan were defined a priori and were not modified after registration. All procedures were performed in accordance with the Declaration of Helsinki (1964) and its later amendments (Brazil, 2013). Written informed consent was obtained from all participants prior to enrollment.

This multi-arm randomized clinical trial was reported in accordance with the CONSORT 2010 Statement and the CONSORT extension for multi-arm parallel-group randomized trials.

### Sample size calculation

An a priori sample size calculation was performed in accordance with the prespecified primary outcome and time point. The primary endpoint was postoperative pain measured on a 10-cm visual analog scale (VAS) at 6 h. The trial was planned as a 2 × 3 factorial design (NaOCl concentration: 2.5% vs. 5.25%; activation: NA vs. EDDY vs. PUI), and the sample size was determined to detect the main effects of concentration and activation at the primary time point (interaction effects were considered secondary/exploratory). Power analysis was conducted using G*Power (ver. 3.1.9.2; Franz Faul, University of Kiel, Germany) with a two-sided α = 0.05 and power = 0.80. A moderate standardized effect size (Cohen’s f = 0.25) was assumed basis of the magnitude and variability of early postoperative pain outcomes reported in previous randomized trials evaluating different NaOCl concentrations [8]. Because no pilot data were available, this value was considered an appropriate and conservative estimate for the sample size calculation. With these assumptions, the required total sample size was 120 participants, corresponding to 20 participants per group (six groups). Secondary pain time points and analgesic intake were analyzed as secondary outcomes and were not used for the sample size calculation.

### Preoperative assessment

At enrollment, a standardized preoperative evaluation was performed. Baseline pain was recorded immediately before treatment using a 10-cm visual analog scale (VAS). Pulp status and periapical findings were assessed using routine clinical diagnostic procedures (sensibility testing and clinical examination), and periapical radiographs were obtained to document the periapical status. Although the presence of chronic apical periodontitis was confirmed radiographically, lesion severity was not standardized using the periapical index (PAI) or another validated radiographic scoring system. A baseline periodontal screening was also performed as part of the eligibility assessment. Only patients meeting the predefined inclusion criteria and none of the exclusion criteria were included.

### Inclusion criteria

- Systemically healthy individuals aged 18–60 years.
- Patients diagnosed with asymptomatic chronic apical periodontitis in three - canal maxillary or mandibular first or second molars, with negative responses to electric pulp testing and cold testing and negative findings on percussion and palpation.
- Patients requiring primary endodontic treatment of a permanent maxillary or mandibular molar, with no preoperative pain at baseline (VAS = 0) recorded immediately prior to treatment, and who provided written informed consent.
- Radiographic confirmation of chronic apical periodontitis on standardized periapical radiographs, defined as the presence of periapical radiolucency consistent with apical periodontitis. A standardized scoring system (e.g., PAI) or lesion size threshold was not applied during the eligibility assessment.
- The absence of clinical signs of acute infection, including no intraoral/extraoral swelling, no sinus tract, and no suppuration at the time of enrollment.

### Exclusion criteria

- Patients who had any preoperative pain at baseline (VAS > 0) and other conditions that could confound postoperative pain assessment (e.g., acute apical abscess/swelling).
- Mandibular and maxillary first or second molars with four or more root canals upon access cavity preparation or radiographic examination.
- Mandibular and maxillary first or second molars with poor periodontal condition (periodontal probing depth > 4 mm) identified at baseline periodontal screening (excluded to minimize confounding from periodontal disease).
- Pregnant or breastfeeding individuals.
- Patients with uncontrolled hypertension, uncontrolled diabetes mellitus, chronic renal failure, hematological disorders, or those receiving chemotherapy or radiotherapy.
- Patients with infective endocarditis, immunosuppressive disorders, or those requiring antibiotic prophylaxis prior to the procedure due to any medical condition.
- Patients who had taken anti-inflammatory or analgesic medications within 24 h before the procedure or who had a history of systemic antibiotic use within the past month were excluded from the study.

### Randomization

**Fig. 1:**
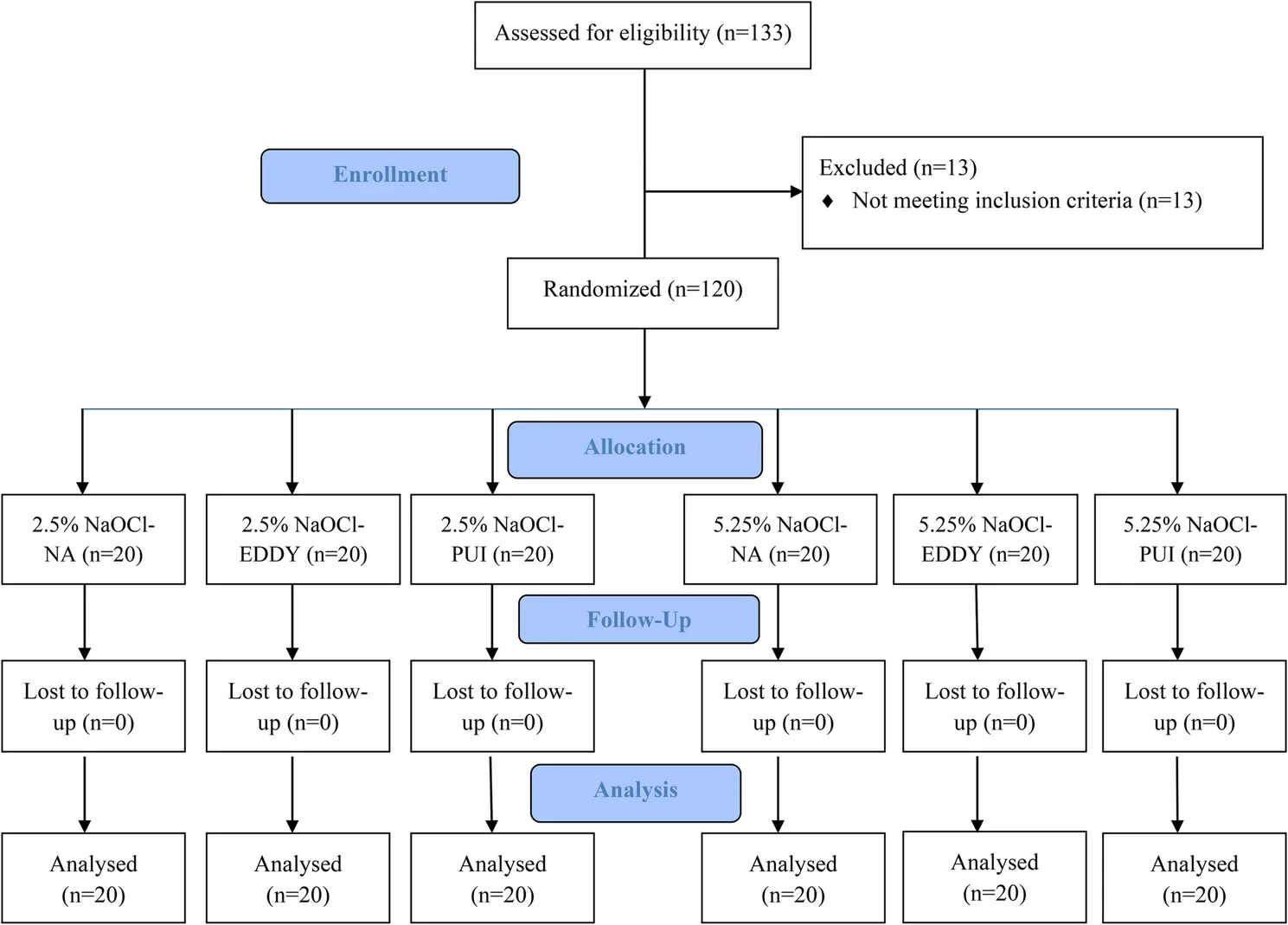
CONSORT flow diagram showing participant enrollment, allocation, follow-up, and analysis

Randomization was performed using a simple random allocation sequence generated with the online randomization tool Randomizer.org (https://www.randomizer.org) before participant enrollment. The allocation sequence was prepared by an investigator who was not involved in participant recruitment, treatment, or outcome assessment. Allocation concealment was ensured by transferring the Randomizer.org-generated allocation sequence to identical folded slips, which were placed in an opaque container. After confirming eligibility and obtaining written informed consent, a dental assistant who was not involved in eligibility assessment, participant recruitment, treatment, or outcome assessment implemented the allocation by drawing one folded slip for each consecutively enrolled participant. The assignment was revealed only after the slip was drawn, thereby maintaining allocation concealment until group assignment (Fig. 1).

### Root canal treatment protocol

Detailed medical histories were obtained from all patients, and their demographic data were recorded. All root canal treatments and irrigation/activation procedures were performed by a single experienced endodontist (E.K.) following a standardized written protocol. This approach was used to minimize operator-related variability. To prevent gingival discomfort caused by the rubber dam clamps, superficial infiltrative anesthesia was administered with 4% articaine hydrochloride containing 1:100,000 epinephrine (Ultracain D-S; Sanofi, Paris, France). After rubber dam isolation was achieved (Coltene/Whaledent; Altstätten, Switzerland), access cavities were prepared via sterile diamond burs. The working length of each canal was determined using an electronic apex locator (Woodpex III; Woodpecker, China) and confirmed via periapical radiographs.

Following apical patency verification with a #10 K-file (Dentsply Sirona; Ballaigues, Switzerland), instrumentation was used as described. Apical patency was maintained during instrumentation with a #10 K-file; however, the patency file was not used after final irrigation or immediately before obturation. The mesiobuccal and mesiolingual canals of mandibular molars were prepared using the Reciproc Blue R25 file (25/0.08; VDW, Munich, Germany), whereas the distal canal was prepared using the Reciproc Blue R40 file (40/0.06). In maxillary molars, the mesiobuccal and distobuccal canals were prepared using the Reciproc Blue R25 file, whereas the palatal canal was prepared using the Reciproc Blue R40 file. Baseline canal diameter was not used as a stratification parameter; however, the instrumentation protocol was standardized according to canal configuration to ensure procedural consistency across all groups.

During the preparation of each root canal, the coronal, middle, and apical thirds were irrigated sequentially using a 30-gauge side-vented irrigation needle (Perfect; Neo Dental International, Seoul, South Korea) with 2 mL of NaOCl (Imicryl; Konya, Türkiye) at the assigned concentration. Thus, a total of 6 mL of NaOCl was delivered per canal during instrumentation. Following instrumentation, a final irrigation step was performed using an additional 6 mL of NaOCl, activated via one of three irrigation techniques: NA, EDDY, or PUI. In total, each root canal received 12 mL of NaOCl throughout the irrigation protocol.

### Activation protocols

#### 2.5% NaOCl – NA Group (*n* = 20)

After the root canal was prepared, final irrigation of each canal was performed using 6 mL of 2.5% NaOCl, which was delivered 2 mm short of the working length with a 30-gauge side-vented needle. Each canal was subsequently irrigated with 2 mL of saline, followed by 2 mL of 17% ethylenediaminetetraacetic acid (EDTA; Imicryl, Konya, Türkiye). EDTA was delivered passively in all groups without activation. To remove the EDTA, an additional 2 mL of saline was used to complete the irrigation procedure.

#### 2.5% NaOCl – EDDY Group (*n* = 20)

Following root canal preparation, final irrigation of each canal was performed using 2.5% NaOCl, which was activated with an EDDY tip (25/0.04) positioned 2 mm short of the working length. Activation was carried out in three cycles, with each cycle involving 2 mL of NaOCl activated for 20 s. Each canal was subsequently irrigated with 2 mL of saline, followed by 2 mL of 17% EDTA. To remove the EDTA, an additional 2 mL of saline was used to complete the irrigation procedure.

#### 2.5% NaOCl – PUI Group (*n* = 20)

After the root canal was prepared, final irrigation of each canal was performed with 2.5% NaOCl, which was activated with an ultrasonic tip (IRRI S 21/25; VDW, Munich, Germany), positioned 2 mm short of the working length. The activation was performed in three cycles, each involving 2 mL of NaOCl activated for 20 s. Each canal was subsequently irrigated with 2 mL of saline, followed by 2 mL of 17% EDTA. To remove the EDTA, an additional 2 mL of saline was used, thereby completing the irrigation procedure.

#### 5.25% NaOCl – NA Group (*n* = 20)

After the root canal was prepared, final irrigation of each canal was performed with 6 mL of 5.25% NaOCl, which was delivered 2 mm short of the working length using a 30-gauge side-vented needle. Each canal was subsequently irrigated with 2 mL of saline, followed by 2 mL of 17% EDTA. To remove the EDTA, an additional 2 mL of saline was used to complete the irrigation procedure.

#### 5.25% NaOCl – EDDY Group (*n* = 20)

Following the completion of root canal preparation, final irrigation of each canal was performed with 5.25% NaOCl, activated with an EDDY tip (25/04), positioned 2 mm short of the working length. Activation was carried out in three cycles, with each cycle involving 2 mL of NaOCl activated for 20 s. Each canal was subsequently irrigated with 2 mL of saline, followed by 2 mL of 17% EDTA. To remove the EDTA, an additional 2 mL of saline was used to complete the irrigation procedure.

#### 5.25% NaOCl – PUI Group (*n* = 20)

After the root canal was completed, final irrigation of each canal was performed with 5.25% NaOCl, which was activated with an ultrasonic tip, positioned 2 mm short of the working length. Activation was carried out in three cycles, with each cycle involving 2 mL of NaOCl activated for 20 s. Each canal was subsequently irrigated with 2 mL of saline, followed by 2 mL of 17% EDTA. To remove the EDTA, an additional 2 mL of saline was used to complete the irrigation procedure.

After the completion of root canal preparation and final irrigation protocols were completed in all groups, the canals were dried with paper points (VDW; Munich, Germany). Root canal obturation was performed by the lateral condensation technique with gutta-percha (VDW; Munich, Germany) and an epoxy resin-based sealer (AH Plus; Dentsply, Konstanz, Germany). The canal orifices were sealed with resin-modified glass ionomer cement (EQUIA Forte; GC, Tokyo, Japan), after which the coronal restorations were completed with composite resin (Z350; 3 M ESPE, Sumare, SP, Brazil), and occlusal adjustments were made. Occlusal adjustments were performed after restoration to eliminate premature contact and ensure standardized occlusal conditions across all treated teeth.

Periapical radiographs were taken following the procedure to evaluate the radiographic adaptation of the root canal fillings to the apex. Postoperative pain intensity was assessed using a 100-mm (10-cm) visual analog scale (VAS). The scale ranged from 0 (“no pain”) to 10 (“worst imaginable pain”), with numbered reference points displayed along the 10-cm line. Patients recorded VAS scores at 6, 12, 24, 48, and 72 h after treatment. Postoperative pain scores were self-reported by the patients using VAS forms and were collected and verified by a blinded investigator who was not involved in the treatment procedures. Participants were instructed to use 400 mg of ibuprofen as a rescue analgesic only if needed. The maximum permitted intake was 1 tablet per dose, which was repeated no more frequently than every 4 h, with a maximum of 6 tablets (total [6 × 400] mg) per 24 h. If pain was not adequately controlled despite the maximum allowable dose, patients were instructed to contact the clinic for further evaluation and management. Rescue analgesic intake (yes/no and total number of tablets) was recorded and analyzed as a secondary outcome. Patients were recalled for clinical follow-up three days after treatment; completed VAS forms were collected and verified by a blinded investigator who was not involved in treatment delivery.

### Statistical analysis

Statistical analyses were performed using IBM SPSS Statistics version 26 (IBM. Armonk, NY, USA). Postoperative pain (VAS) measured at 6, 12, 24, 48, and 72 h was analyzed using a linear mixed-effects model to account for repeated measurements within participants and the 2 × 3 factorial design (NaOCl concentration: 2.5% vs. 5%; NA vs. activation technique: EDDY and PUI). Fixed effects included concentration, activation technique, time, and prespecified interaction terms (concentration × activation, time × concentration, and time × activation). Age was included as a covariate to address baseline imbalance. Correlations among repeated measures within participants were modeled using a first-order autoregressive [AR(1)] covariance structure, as this structure assumes that correlations between repeated observations decrease as the time interval between measurements increases, which was considered appropriate for postoperative pain assessments performed at sequential follow-up time points.

Because the activation × time interaction was significant, prespecified simple effects at the primary time point (6 h) were evaluated, with pairwise comparisons adjusted using the Bonferroni correction. Rescue analgesic intake (yes/no) was analyzed as a secondary outcome using patient-level binary logistic regression (one record per participant), including concentration and activation technique as categorical predictors and age as a covariate; the results are presented as odds ratios (OR) with 95% confidence intervals. Statistical significance was set at α = 0.05. Standardized effect sizes for the fixed effects in the linear mixed-effects model are reported as partial eta squared (η²p).

The assumptions of the linear mixed-effects model were assessed by examining the normality of the residuals and the homogeneity of residual variance. No major deviations from these assumptions were observed.

## Results

The demographic characteristics of the participants, including age, sex, and tooth location, are presented in Table 1. Baseline age differed significantly across the six study groups (one-way ANOVA, F(5,114) = 3.164, *P* = .010), whereas no statistically significant between-group differences were observed for sex (χ²(5) = 8.038, *P* = .154) or tooth location (χ²(5) = 0.686, *P* = .984). Therefore, age was included as a prespecified covariate in the primary model-based analyses of postoperative pain and analgesic intake.

**Table 1:** Baseline characteristics of participants by study group

Study Groups	2.5% NaOCl–NA (*n* = 20)	2.5% NaOCl–EDDY (*n* = 20)	2.5% NaOCl–PUI (*n* = 20)	5.25% NaOCl–NA (*n* = 20)	5.25% NaOCl–EDDY (*n* = 20)	5.25% NaOCl–PUI (*n* = 20)	Total (*n* = 120)	*P* value
Age (mean ± SD)	32.3 ± 14.95	32.6 ± 14.51	28.7 ± 13.91	31.8 ± 14.73	25.2 ± 8.81	20.0 ± 5.31	28.43 ± 13.14	**0.010**
Gender								
Female, n (%)	12 (60)	18 (90)	12 (60)	12 (60)	10 (50)	12 (60)	76 (63.3)	0.154
Male, n (%)	8 (40)	2 (10)	8 (40)	8 (40)	10 (50)	8 (40)	44 (36.7)	
Tooth type								
Maxillary molar, n (%)	8 (40)	8 (40)	10 (50)	8 (40)	8 (40)	8 (40)	50 (41.7)	0.984
Mandibular molar, n (%)	12 (60)	12 (60)	10 (50)	12 (60)	12 (60)	12 (60)	70 (58.3)	

Continuous variables were compared using one-way analysis of variance (ANOVA), and categorical variables were compared using Pearson’s chi-square test. Age differed significantly among the study groups (*P* = .010); therefore, age was included as a prespecified covariate in the primary model-based analyses. No significant between-group differences were observed for gender or tooth type

**Fig. 2:**
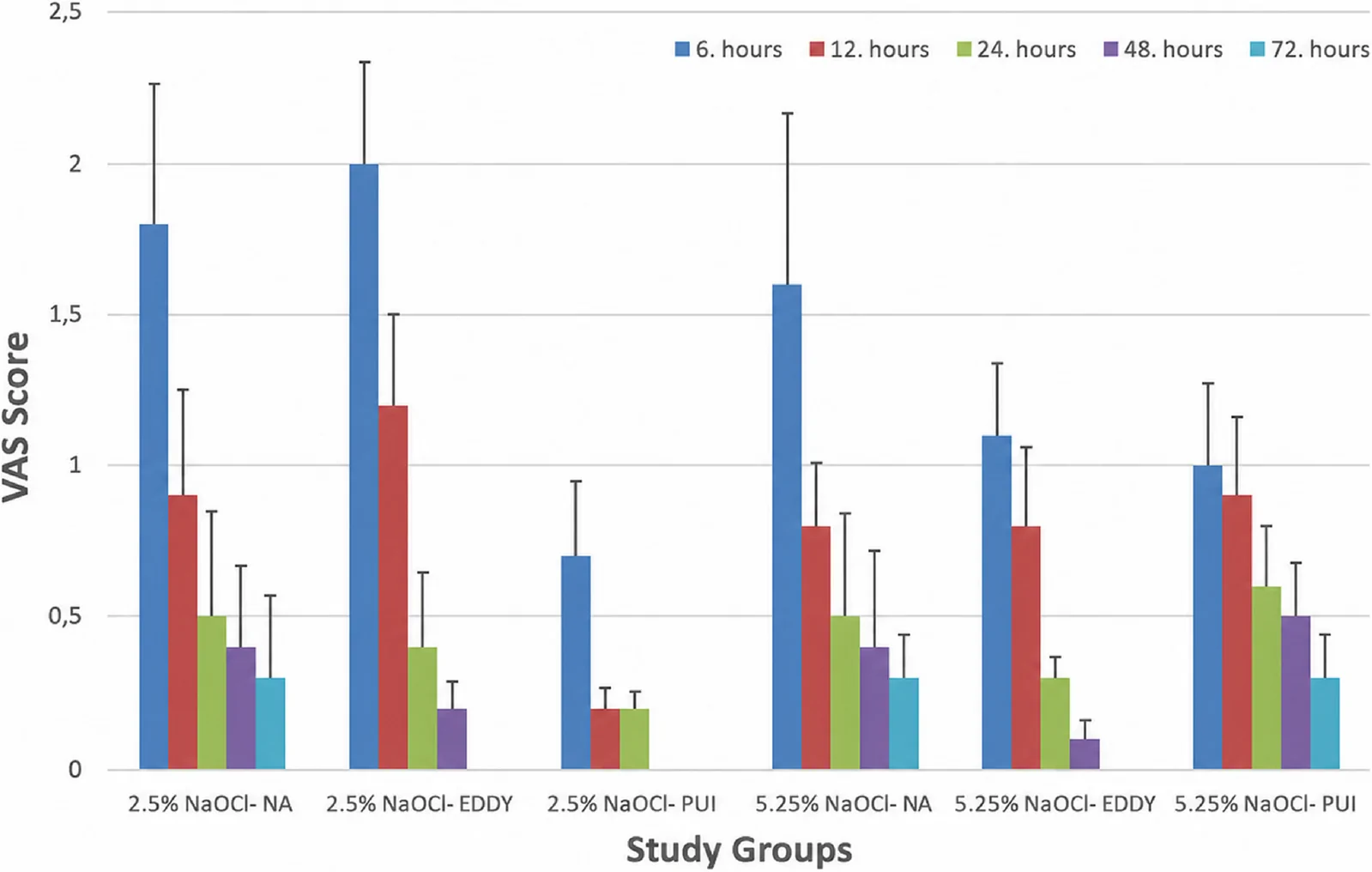
Mean postoperative pain scores (VAS) at 6, 12, 24, 48, and 72 h in the six study groups according to NaOCl concentration and activation technique. Bars represent mean VAS scores, and error bars indicate standard deviations (SD). Groups: 2.5% NaOCl–NA, 2.5% NaOCl–EDDY, 2.5% NaOCl–PUI, 5.25% NaOCl–NA, 5.25% NaOCl–EDDY, and 5.25% NaOCl–PUI. Abbreviations: NA, nonactivation; PUI, passive ultrasonic irrigation; VAS, visual analog scale; NaOCl, sodium hypochlorite

Postoperative pain trajectories over time are summarized in Table 2; Fig. 2. In the overall linear mixed-effects model (AR(1) covariance structure) evaluating repeated VAS measurements at 6, 12, 24, 48, and 72 h, significant effects were observed for activation technique (F (2,122.922) = 4.646, *P* = .011, η²*p* = .070) and time (F (4,413.154) = 26.507, *P* < .001, η²*p* = .204), and age was also significantly associated with pain (F (1,125.951) = 18.683, *P* < .001, η²*p* = .129). The main effect of NaOCl concentration was not significant (F (1,122.972) = 0.398, *P* = .529, η²*p* = .003). Significant interactions were detected for concentration × activation (F (2,125.951) = 3.392, *P* = .037, η²*p* = .051) and activation × time (F (8,413.154) = 5.762, *P* < .001, η²*p* = .100), whereas concentration × time was not significant (F (4,413.154) = 0.862, *P* = .487, η²*p* = .008) (Table 3, Panel A).

**Table 2:** Postoperative pain scores (VAS) by group and time point

	2.5% NaOCl - NA	2.5% NaOCl - EDDY	2.5% NaOCl - PUI	5.25% NaOCl - NA	5.25% NaOCl - EDDY	5.25% NaOCl - PUI
	mean ± SD / median (min-max)	mean ± SD / median (min-max)	mean ± SD / median (min-max)	mean ± SD / median (min-max)	mean ± SD / median (min-max)	mean ± SD / median (min-max)
6. hours	1.7 ± 0.46 / 1 (0–6)	2 ± 0.34 / 2 (0–5)	0.7 ± 0.25 / 0 (0–3)	1.6 ± 0.57 / 0.5 (0–6)	1.1 ± 0.24 / 0 (0–3)	1 ± 0.27 / 0.5 (0–3)
12. hours	0.9 ± 0.35 / 0 (0–5)	1.2 ± 0.30 / 1 (0–4)	0.2 ± 0.07 / 0 (0–1)	0.8 ± 0.21 / 0 (0–3)	0.8 ± 0.26 / 0 (0–3)	0.9 ± 0.26 / 0.5 (0–3)
24. hours	0.5 ± 0.35 / 0 (0–5)	0.4 ± 0.15 / 0 (0–2)	0.2 ± 0.06 / 0 (0–1)	0.5 ± 0.34 / 0 (0–5)	0.3 ± 0.07 / 0 (0–1)	0.6 ± 0.20 / 0.5 (0–2)
48. hours	0.4 ± 0.27 / 0 (0–4)	0.2 ± 0.09 / 0 (0–1)	0 ± 0 / 0 (0–0)	0.4 ± 0.32 / 0 (0–5)	0.1 ± 0.06 / 0 (0–1)	0.5 ± 0.18 / 0 (0–2)
72. hours	0.3 ± 0.27 / 0 (0–4)	0 ± 0 / 0 (0–0)	0 ± 0 / 0 (0–0)	0.3 ± 0.14 / 0 (0–2)	0 ± 0 / 0 (0–0)	0.3 ± 0.14 / 0 (0–2)

Values are presented as mean ± standard deviation (SD) and median (minimum–maximum) to descriptively summarize postoperative pain scores. Inferential comparisons were based on estimated marginal means derived from the linear mixed-effects model and are reported in the text and Table 3

**Table 3:** Model-based effects on postoperative pain and primary time-point contrasts

Panel A. Linear mixed-effects model (AR (1)) — Type III tests of fixed effects					
Effect	Numerator df	Denominator df	F	* P* value	Partial η²p
NaOCl concentration(Conc)	1	122.972	0.398	.529	.003
Activation technique(Act)	2	122.922	4.646	.011	.070
Time	4	413.154	26.507	< .001	.204
Age	1	125.951	18.683	< .001	.129
Conc × Act	2	125.951	3.392	.037	.051
Time × Conc	4	413.154	0.862	.487	.008
Time × Act	8	413.154	5.762	< .001	.100

Panel B. Primary time point (6 hours) — activation simple effects and Bonferroni-adjusted contrasts					
Activation technique	Estimated marginal mean (6 h)	95% CI			
NA	2.160	1.671 - 2.649			
EDDY	1.427	0.945 - 1.909			
PUI	0.613	0.122 - 1.104			

Contrast	Mean difference	95% CI	Bonferroni *P*		
NA − EDDY	0.733	-0.136 - 1.603	.109		
NA − PUI	1.546	0.683 - 2.410	< .001		
EDDY − PUI	0.813	-0.056 - 1.682	.063		

Panel A reports Type III tests from the linear mixed-effects model with an AR(1) covariance structure; age was included as a covariate. Partial eta squared (η²p) values are reported as standardized effect-size estimates for the fixed effects

Panel B reports prespecified simple effects at 6 hours; pairwise contrasts are Bonferroni-adjusted.NA, nonactivation; PUI, passive ultrasonic irrigation; CI, confidence interval

Given the significant activation × time interaction, prespecified simple effects were examined at the primary time point (6 h) with Bonferroni adjustment. At 6 h, the activation technique significantly influenced pain (F (2,113) = 9.479, *P* < .001). The estimated marginal means at 6 h were 2.160 for NA (95% CI 1.671–2.649), 1.427 for EDDY (95% CI 0.945–1.909), and 0.613 for PUI (95% confidence interval (CI) 0.122–1.104). Bonferroni-adjusted pairwise comparisons indicated significantly greater pain for NA than for PUI (mean difference 1.546; 95% CI 0.683–2.410; *P* < .001), whereas NA versus EDDY (*P* = .109) and EDDY versus PUI (*P* = .063) were not significant after adjustment (Table 3, Panel B). At 6 h, the concentration × activation interaction remained significant (F (2,113) = 4.098, *P* = .019), whereas the main effect of concentration was not significant (F (1,113) = 3.268, *P* = .073) (Table 3, Panel B).

Rescue analgesic intake is presented in Table 4. Overall, 22/120 participants (18.3%) reported analgesic intake. In the patient-level logistic regression adjusted for age, the NaOCl concentration was not associated with analgesic intake (OR = 0.982; 95% CI 0.369–2.619; *P* = .972). With PUI as the reference, EDDY was associated with higher odds of analgesic intake (OR = 4.022; 95% CI 1.097–14.748; *P* = .036), whereas NA versus PUI was not statistically significant (OR = 3.364; 95% CI 0.879–12.876; *P* = .077). Age was inversely associated with analgesic intake (OR = 0.939 per year; 95% CI 0.893–0.987; *P* = .014) (Table 4, Panel B).

**Table 4:** Analgesic intake by group and adjusted association with study factors

Panel A. Analgesic intake by group (n/N, %)							
	2.5% NaOCl - NA	2.5% NaOCl - EDDY	2.5% NaOCl - PUI	5.25% NaOCl - NA	5.25% NaOCl - EDDY	5.25% NaOCl - PUI	Total
	(n=20)	(n=20)	(n=20)	(n=20)	(n=20)	(n=20)	(n=120)
Analgesic intake							
Yes	2 (10)	8 (40)	0 (0)	6 (30)	2 (10)	4 (20)	22 (18.3)
No	18 (90)	12 (60)	20 (100)	14 (70)	18 (90)	16 (80)	98 (81.7)

Panel B. Patient-level logistic regression (dependent variable: analgesic intake Yes=1)							
Predictor	Adjusted OR	95% CI	*P *value				
NaOCl concentration	.982	0.369 - 2.619	.972				
(5.25% vs 2.5%)							
Activation: NA vs PUI	3.364	0.879 - 12.876	.077				
(reference)							
Activation: EDDY vs PUI	4.022	1.097 - 14.748	.036				
(reference)							
Age	.939	0.893 - 0.987	.014				
(per 1-year increase)							

Panel A values are n (%). Panel B values are adjusted odds ratios (OR) with 95% confidence intervals

Reference categories: concentration = 2.5% NaOCl; activation = PUI

OR > 1 indicates higher odds of analgesic intake. Age is modeled continuously

## Discussion

Postoperative pain following root canal treatment is a condition that can significantly impair patients’ quality of life after the procedure, and addressing this issue promptly is crucial for both patient satisfaction and clinical success [15]. Postoperative pain primarily results from inflammatory responses in periapical tissues mediated by pro-inflammatory cytokines and neuropeptides released following tissue injury or microbial stimulation, which increase vascular permeability and sensitize peripheral nerve endings, thereby intensifying pain perception [16].

Among the mechanical factors influencing postoperative pain, procedural errors during root canal shaping and irrigation are particularly prominent. Moreover, the low biocompatibility and cytotoxicity of certain irrigating solutions, together with the apical extrusion of irrigants or intracanal debris during irrigation activation, may exacerbate the inflammatory response and contribute to postoperative pain [17, 18]. Although the use of NaOCl at higher concentrations can improve tissue dissolution and antimicrobial efficacy, its extrusion beyond the apex can irritate surrounding tissues and lead to increased postoperative pain [8]. In addition to irrigation-related factors, instrumentation kinematics may also influence postoperative pain. Although previous studies have reported inconsistent findings regarding the influence of instrumentation motion on postoperative pain, several studies have suggested that reciprocating instrumentation may be associated with greater postoperative pain than continuous rotary instrumentation, possibly because of increased apical extrusion of dentinal debris and microorganisms [19]. Nevertheless, all canals in the present study were prepared using the same reciprocating file system under a standardized protocol. Therefore, any potential influence of instrumentation kinematics was consistent across all study groups and is unlikely to have affected the between-group comparisons. This factor should nevertheless be considered when interpreting the findings.

To our knowledge, evidence directly evaluating, within a single factorial framework, the combined effects of NaOCl concentration and irrigant activation on postoperative pain in molars with chronic apical periodontitis is limited. Therefore the present study investigated whether 2.5% versus 5.25% NaOCl, combined with NA, EDDY, or PUI influences postoperative pain following single-visit root canal treatment of molars diagnosed with chronic apical periodontitis.

Previous clinical studies have evaluated various irrigation activation techniques in relation to postoperative pain [20–24]. In accordance with these studies, EDDY and PUI were selected as representative sonic and ultrasonic activation techniques.

We enrolled asymptomatic patients because preoperative pain is a strong predictor of postoperative pain intensity [25, 26] and could confound the interpretation of intervention-related effects. Standardizing baseline pain to VAS = 0 improves internal validity for isolating effects attributable to irrigation parameters, but it also means that absolute postoperative pain levels may be low, potentially introducing a floor effect. In addition, to reduce anatomical heterogeneity and standardize clinical conditions, only mandibular first and second molars with three canals and maxillary first and second molars without an MB2 canal were included. Although this approach improved standardization, it may limit the generalizability of the findings to maxillary molars presenting with an MB2 canal. Periapical pathology may also influence postoperative pain through factors such as the microbial load, inflammatory status, and apical tissue sensitivity. However, lesion size was not quantitatively assessed in the present study; therefore, its potential contribution to postoperative pain could not be evaluated and should be considered when the findings are interpreted.

Pain perception is a subjective experience that varies among individuals and can be influenced by factors such as age, sex, and socioeconomic status. In clinical research, the VAS is typically used to assess pain quantitatively, as it facilitates patients’ numerical expression of pain intensity [27]. In the present study, postoperative pain scores were recorded via the VAS at 6, 12, 24, 48, and 72 h following treatment.

In the prespecified linear mixed-effects model accounting for repeated measures and the 2 × 3 factorial design (with age adjustment), postoperative pain decreased over time across all groups, which is consistent with the expected postoperative course. Although the NaOCl concentration alone was not associated with postoperative pain, the activation technique influenced early postoperative pain, and its effect varied over time. Because a significant concentration × activation interaction was observed, the effect of NaOCl concentration was interpreted in the context of the activation technique rather than in isolation. Accordingly, the null hypothesis for NaOCl concentration was not rejected, whereas those for the activation technique and the concentration × activation interaction were rejected. These findings indicate the that NaOCl concentration and activation technique should be considered together when their potential influence on postoperative pain is interpreted. It is plausible that activation enhances the effectiveness of lower NaOCl concentrations by improving irrigant penetration, fluid dynamics, and exchange within the root canal system. Despite the statistically significant differences observed between groups, statistical significance does not necessarily indicate that the differences exceeded the minimal clinically important difference perceived by patients. Because no universally accepted minimal clinically important difference has been established specifically for postoperative endodontic pain, the findings should also be interpreted in relation to the absolute magnitude of the observed differences. In the present study, between-group differences at 6 h were modest, and postoperative pain scores remained low overall, suggesting that the clinical impact of the activation-related differences may be limited.

In a clinical study conducted by Paixão et al. [23] on 80 single-rooted teeth diagnosed with either irreversible pulpitis or pulp necrosis, researchers compared 5% NaOCl activated using EDDY and manual dynamic activation. They reported significantly higher pain scores in the EDDY group during the early postoperative period. The authors attributed these findings to the high concentration of NaOCl and the potential for increased apical extrusion of debris associated with the high-frequency oscillatory motion of the EDDY tip, which may trigger an inflammatory response in the surrounding tissues and thereby intensify postoperative pain. Differences in tooth type, particularly single-rooted teeth versus molars, and pulp diagnosis, including the presence of irreversible pulpitis in the previous study, may explain the discrepancies between their findings and the present results. In contrast, Gündoğar et al. [20] evaluated the effect of 3% NaOCl activated by NA, EDDY, EndoActivator, and PUI on postoperative pain in mandibular premolars with symptomatic irreversible pulpitis and reported no statistically significant differences among the groups. These discrepancies may reflect differences in tooth type, pulpal diagnosis, and irrigation protocols. We speculate that the findings of the present study may be related to the high-frequency sonic oscillation generated by the EDDY tip together with its 0.4 taper tip configuration, which may increase apical debris extrusion compared with PUI.

NaOCl remains the most widely used irrigant because of its antimicrobial activity and tissue-dissolving properties; however, its cytotoxicity and low biocompatibility may contribute to adverse periradicular responses if extrusion occurs [28, 29]. Its clinical performance depends on multiple factors, including concentration, volume, temperature, and delivery or activation conditions [7, 30–32]. In the present study, the NaOCl concentration alone (2.5% versus 5.25%) was not associated with postoperative pain, which is consistent with the findings of several randomized clinical studies reporting no clear concentration-dependent differences across commonly used NaOCl concentrations [33–37]. In contrast, Farzaneh et al. [28] reported lower postoperative pain with 5.25% NaOCl in symptomatic molars, suggesting that differences in pulpal diagnosis and study methodology may explain these inconsistent findings. In the present study, the total NaOCl volume was standardized at 12 mL per canal to minimize variability in irrigant quantity and isolate the effects of concentration and activation. However, the use of relatively high, consistent irrigant volumes may also reduce detectable differences between concentrations by ensuring adequate irrigant exchange and tissue dissolution even at lower concentrations. Therefore, the absence of a concentration effect should be interpreted in the context of the standardized irrigation volume and the significant interaction between the NaOCl concentration and activation technique.

Analgesic intake was analyzed as a secondary outcome using patient-level logistic regression to avoid multiple unplanned group comparisons. After adjustment for age, the NaOCl concentration was not associated with analgesic intake. When PUI was used as the reference, EDDY was associated with higher odds of analgesic intake, whereas the difference between NA and PUI did not reach conventional statistical significance. The confidence intervals were wide, likely reflecting the relatively small number of participants who required rescue analgesics and the resulting uncertainty in the estimated odds ratios. Therefore, these secondary findings should be interpreted cautiously and in conjunction with the early postoperative pain pattern. Although apical extrusion was not directly measured in this study, differences in tip design, oscillation pattern, and intracanal hydrodynamics between the sonic and ultrasonic activation systems may have contributed to differences in postoperative pain and analgesic intake.

Although statistically significant differences were observed during the early postoperative period, absolute postoperative pain scores remained low. Therefore, optimizing irrigant activation protocols may have a greater influence on early postoperative discomfort than increasing the NaOCl concentration alone does, although the clinical relevance of these differences should be interpreted cautiously.

This study contributes evidence on the combined evaluation of irrigant concentration and activation technique in a clinically challenging scenario (single-visit treatment of molars with chronic apical periodontitis). However, several limitations should be acknowledged. First, the multi-arm design reduces per-group power and increases the likelihood of chance imbalances; a statistically significant age imbalance was observed and addressed by including age as a covariate. Second, enrolling only asymptomatic three-canal molars improved internal validity but may limit the external validity and generalizability of the findings and may also have contributed to floor effects. Third, operator blinding was not feasible because of the nature of the activation devices; however, outcome assessment was performed independently. In addition, smoking and other behavioral factors were not recorded and therefore could not be adjusted for. Although randomization reduces confounding on average, residual confounding cannot be excluded. Periapical lesion severity was not standardized using the PAI or another validated radiographic scoring system, as these measures were not incorporated into the original study design. Consequently, the potential influence of lesion severity on postoperative pain could not be evaluated. Another limitation is that the trial was registered after participant recruitment had begun. Although the study design, eligibility criteria, outcomes, and statistical analysis plan were predefined and remained unchanged after registration, prospective trial registration is considered the preferred standard for randomized clinical trials and should be adopted in future studies.

## Conclusion

Within the limitations of this randomized clinical trial, postoperative pain after single-visit endodontic treatment of asymptomatic molars with chronic apical periodontitis was more closely associated with the irrigation activation modality than with the NaOCl concentration alone. These findings support the clinical relevance of selecting activation protocols that improve irrigant effectiveness while minimizing early postoperative discomfort.

## Data Availability

All data generated or analysed during this study are included in this published article.

## Abbreviations

NA: Non-Activation
PUI: Passive Ultrasonic Irrigation
NaOCl: Sodium Hypochlorite
EDTA: Ethylene Diamine Tetra Acetic Acid
VAS: Vısual Analog Scale
